# Spectral Knowledge (SK-UTALCA): Software for Exploratory Analysis of High-Resolution Spectral Reflectance Data on Plant Breeding

**DOI:** 10.3389/fpls.2016.01996

**Published:** 2017-01-09

**Authors:** Gustavo A. Lobos, Carlos Poblete-Echeverría

**Affiliations:** ^1^Plant Breeding and Phenomic Center, Facultad de Ciencias Agrarias, PIEI Adaptación de la Agricultura al Cambio Climático, Universidad de TalcaTalca, Chile; ^2^Escuela de Agronomía, Pontificia Universidad Católica de ValparaísoQuillota, Chile; ^3^Department of Viticulture and Oenology, Faculty of AgriSciences, Stellenbosch UniversityMatieland, South Africa

**Keywords:** phenotyping, phenomic, scan, wavelength, noise, outlier, spectral reflectance index (SRI), collinearity

## Abstract

This article describes public, free software that provides efficient exploratory analysis of high-resolution spectral reflectance data. Spectral reflectance data can suffer from problems such as poor signal to noise ratios in various wavebands or invalid measurements due to changes in incoming solar radiation or operator fatigue leading to poor orientation of sensors. Thus, exploratory data analysis is essential to identify appropriate data for further analyses. This software overcomes the problem that analysis tools such as Excel are cumbersome to use for the high number of wavelengths and samples typically acquired in these studies. The software, Spectral Knowledge (SK-UTALCA), was initially developed for plant breeding, but it is also suitable for other studies such as precision agriculture, crop protection, ecophysiology plant nutrition, and soil fertility. Various spectral reflectance indices (SRIs) are often used to relate crop characteristics to spectral data and the software is loaded with 255 SRIs which can be applied quickly to the data. This article describes the architecture and functions of SK-UTALCA and the features of the data that led to the development of each of its modules.

## Introduction

The responses of any living organism are ultimately controlled by genes (G), but the expression of these are modulated in several ways, partly because of the action of other genes, and the complex interaction between them, but mostly in response to the environment (E) where the plant grows and develops (GxE interaction). Gene sequencing is becoming more routine, economical, and fast, but for proper analysis and interpretation of the information an adequate phenotypic characterization is essential, even though it poses one of the greatest difficulties (Lörz and Wenzel, 2005; Finkel, 2009; Lobos et al., 2014; Estrada et al., 2015).

Progress in science and technology have made it possible to study different processes involved in multiple areas of knowledge. In agronomy and biological sciences, sensors, and instrumentation have been developed to characterize the behavior of a particular organism, or a group of them, under a specific environmental condition or situation.

Currently, equipment, techniques, and analyses are available that have proved helpful in characterizing the phenotype (phenotyping), and in the case of remote sensing, quick and high predictive power (Lobos and Hancock, 2015; Camargo and Lobos, 2016).

Among the available remote sensing tools, spectrometers, or spectroradiometers mainly exploit the principle of quantifying the proportion of reflected radiation by an object relative to the incident radiation (Borengasser et al., 2008). Reflectance (graphically represented by the spectral signature) is related to the absorption and transmission of each wavelength, thus representing plant status under ambient or experimental conditions (Garriga et al., 2014). For example, compared to a senescing plant, a healthy one should absorbs more in the visible (blue and red light) and reflect more in the near infrared range.

Nowadays plant reflectance can be measured from space by satellites (with certain limitations on interpretation due to pixel resolution) or from the troposphere by manned and unmanned aerial vehicles (problems related to the number and resolution of the spectrum bands; Araus and Cairns, 2014). Equipment used on the ground covers a wider range of the spectrum, with a better resolution. The most modern devices not only measure into the near infrared region (700–1300 nm; NIR), but also from the ultraviolet (~200 nm) up to the short wavelength infrared (~2500 nm) (Cabrera-Bosquet et al., 2012). This high-resolution technology allows examination of plants beyond the 1000 nm region of the spectrum, with great potential for phenotype prediction (Garbulsky et al., 2011; White et al., 2012; Araus and Cairns, 2014; Lobos et al., 2014).

Several critical issues for making good reflectance measurements in the field have been reported in the literature (e.g., Curtiss and Goetz, 1994; Milton et al., 1995; Salisbury, 1998; Schaepman, 1998; Curtiss and Goetz, 2001). Independent of the equipment used on the ground, a correct measurement of the reflectance in the field is mandatory. Ideally, measurements should be restricted to clear sky conditions, performing a radiometric calibration every 10–15 min to limit variations in reflectance induced by changes in the angle of the sun, and taking in account basic but important considerations such as maintaining the same orientation, angle, and distance to the canopy on each assessed plot or ensuring dark colored clothing for the operators. Although instrument settings vary among brands and models, a number of steps should be followed to optimized data capture. The equipment should be turned on in advance to allow the device to equilibrate with the ambient temperature, the integration time for a single scan or sample needs to be defined (maximizing sensitivity, but avoiding saturation), the number of scans per sample or samples per plot and the convenience of averaging them before data processing should be determined, and the exact sequence for checking darks and standards recommended by the manufacturer needs to be ascertained.

In general, due to its simplicity and ability to forecast several phenotypic characteristics, reflectance is used to calculate “Spectral Reflectance Indices” (SRIs) (Lobos and Hancock, 2015). SRIs are based on relationships between wavelengths or spectrum bands, usually designed to be relatively immune to changes in solar radiation between measurements, relating them quantitatively to changes in plant phenotype (Mullan, 2012). Today there are hundreds of SRIs proposed to estimate different traits (e.g., leaf area index, yield, gas exchange, fluorescence, pigment content, plant water status, carbon isotopic discrimination, etc.) but because of the lack of tools capable of assessing several SRIs at the same time, most of the published works focus on a small percentage of them (e.g., SR, NDVI, WI, NDWI, PRI, SAVI, etc.).

In breeding programs, there is a need to regularly evaluate hundreds or thousands of genotypes in a short time. Therefore, due to the time and cost involved breeders have not been able to perform a thorough phenotypic characterization of the material, limiting their evaluations to the yield, its components and some others traits that are relatively easy to assess (Kipp et al., 2014; Lobos and Hancock, 2015; Camargo and Lobos, 2016). With the emergence of phenomics, which is the acquisition of high-dimensional phenotypic data (high-throughput phenotyping) for characterization of the phenotype of organisms in a multidimensional manner (Houle et al., 2010; Kipp et al., 2014), measurements that used to take weeks or months can now be performed in a few hours (White et al., 2012; Lobos and Hancock, 2015). The implementation of phenomics in plant breeding programs is relatively new, and is an area where more development is likely to be needed (Lobos and Hancock, 2015).

For a correct interpretation of spectral reflectance data, it is essential to have reliable and representative information, especially when it comes from field measurements. The use of reflectance data in breeding programs has several advantages but probably the major problem is the amount of data originated by the numbers of wavelengths and genotypes assessed. If the reflectance data is analyzed in a conventional way (e.g., Excel files), the detection of measurement errors, the study of the spectral noise (originating from absorption by environmental compounds such as water or CO_2_) or the relationship between a specific wavelength and a response variable become difficult or subjective. Nevertheless, as far as we are aware there is currently no free software available that allows detailed exploratory analysis of high-resolution spectral reflectance data. Therefore, the aim of this article is to present an overview of the architecture and functions of Spectral Knowledge (SK-UTALCA), software that has been specially developed for exploratory analysis of high-resolution spectral reflectance data, with applications in plant breeding research and also in many other fields.

Due to the broad nomenclature related to spectral measurements, some definitions are given in order to facilitate the understanding of this article and software (Table 1).

**Table 1:** **Nomenclature related to spectrometer data collection**.

**In this article**	**In other articles or manuals**	**Definition**
Plot		Land area where a single genotype is growing; in other studies it could be considered as a replication.
Scan	Data collection, spectrum or spectra collection, scanning	Action oriented to collect the spectrum or spectra by one scan or shoot (informal terminology).
Samples	Sample spectra, samples of scan, scanned samples, scanned data, artifacts, features	Some spectrometers are able to register several samples within the same scan; number of spectral signatures captured per scan.
Integrations per sample	Spectrum average or averaging	Integration of spectra within the same sample.

## Main SK-UTALCA architecture and functionalities

Spectral Knowledge (SK-UTALCA) is a software package developed in Matlab® and is available compiled for use in a Windows 64-bit environment from a download link or as source code in supplementary material. This program allows, in an efficient and versatile manner, two types of actions: (i) cleaning of the data matrix by studying the spectral noise, and detecting within- and between- measurement errors; and (ii) application of a preliminary analysis of wavelength collinearity, and the detection of wavelengths or SRIs related to a response variable (Table 2).

**Table 2:** **Main SK-UTALCA functionalities according to the program menu**.

**Main objective**	**Main commands**	**Secondary commands**	**Description**
Input and output of information	Import X and Y data	Spectral data (X)	Import spectral data: first column or row (depends on the equipment) must include the assessed wavelengths.
		Samples per plot	Indicate samples per scan (definitions in Table 1).
		Transpose data	Software works only with wavelengths as columns; the user will be able to transpose their data.
		Response variable (Y)	Import response variables data (on columns) where the three first columns must be codes (free criteria).
	Export data	Average	It is possible to export the average of the samples per scan or each sample individually.
		Empty data	Data can be exported including or excluding cells deleted during the cleaning of the data matrix.
Cleaning data matrix	Noise analysis	Wavelength segments	Ten different segments to analyze in relation to the percentage change among a determined neighbor size.
			Noise elimination can be applied equally to all data (Group) or for each sample (Individual). Additionally, negative values can be also deleted.
	Scan analysis	Maximum variation coefficient	Criteria to select samples within a same scan where the variation coefficient, at any wavelength, is lower than the established threshold (Scans without problems) and those that exceeded it (Scans with problems).
		Samples to delete	If there are inconsistencies in one or more samples within the same scan, it is possible to select and delete them.
	Outlier analysis		Through a graphical analysis of the cloud of data points (response variable vs. SRI), it is possible to detect those out of range, identify the source of the problem and delete them in the case of clear evidence of a mistake.
Preliminary analysis	Collinearity analysis		For a given response variable, through linear or artificial neural network (ANN) analysis, it is possible to identify wavelengths without collinearity.
	Individual wavelength analysis		Through different regression models and statistical parameters, it is possible to identify wavelengths better associated with a given response variable.
	SRI analysis	Full report	Through different regression models and a coefficient of determination threshold, it is possible to identify SRIs that are better associated with a given response variable. The software will be launched with a database of 255 SRIs (Supplementary Table 1).
		Detailed index report	For subsequent graphical representation it is possible to export, for each genotype or measurement, individual values of SRIs and response variables.

After X and Y are loaded, the user can perform any main command, without a specific order. At the same time, each analysis can be run considering the previous exploration (*Run from current data*) or from the original data (loaded as X) (*Run from original data*). On each section of the cleaning data matrix or the preliminary analysis, the user will be able to export the analyzed information (csv format).

### Main menu (data set)

In the main menu, users are required to load the spectral reflectance data [*Spectral data (x)*]. The first step in the use of SK-UTALCA software is to load the data set in Microsoft Excel format (xlsx format). To read the spectral data it is necessary to indicate the number of samples taken in each plot (*Samples per plot*). Depending on the equipment used, reflectance data is organized in columns or rows. In the software, the spectral bands need to be located in columns and the spectral data in rows (it is possible to use the “*Transpose data*” option to relocate the data set as needed). The first wavelength measured must be in the second column (the first column is for codification purposes and depends only on the user).

The second file needed (xlsx format) contains the values for the independent variables [*Response variables (y)*] for each plot. In this case, the spreadsheet must consider three codification columns and the first variable should be allocated to the fourth column.

The software has no limitation on the number of spectral data points (wavelengths or measurements) or response variables.

### Noise analysis

This first filter removes the spectral noise originated by the natural presence of certain elements in the atmosphere, such as water and carbon dioxide, which absorb specific wavelengths (Salisbury, 1998; Curtiss and Goetz, 2001; Psomas et al., 2005; Ma and Chen, 2006; Clevers et al., 2008). Researchers who screen hundreds or thousands of genotypes under field conditions usually consider at least three or four spectral samples per plot, generating a matrix of data that makes it difficult to objectively select the noise segment(s) for deletion. Furthermore, spreadsheet graphical options are usually restricted to a maximum number of data series per chart (e.g., ~255 in Excel for Windows or Mac), so there is no easy way to take a decision based on this tool. For this reason, for breeding purposes, conventional visual noise elimination is not a real alternative, restricting the criteria to the assumption of arbitrary limits, usually following thresholds from a third person or related articles.

With this module, it will be possible to analyze the spectral noise by considering up to ten independent segments. This will allow the user to set up different criteria in each segment, being more or less strict depending on the wavelengths analyzed, the data collected or previous knowledge. To apply the filter on each spectral signature, it is necessary to indicate the lower and upper limit for each segment (*Wavelength segments*), the maximum accepted percentage of variations (%) between two neighboring wavelengths, and the number of neighbors (*N size*) where the previous condition is found consecutively. The graphic window will show red crosses where the first criterion is satisfied and black ones where both have been met, this last condition determining where the software will perform the cleaning.

However, an objective selection is not the only important aspect of spectral noise. During the day there are environmental changes (e.g., relative humidity) that not only affect the magnitude of each problematic wavelength, but also the number of wavelengths involved. For instance, measurements performed under conditions of higher relative humidity (usually before midday) produce wider noise segments beyond 1000 nm; if the determination of the number of wavelengths to eliminate considers measurements across the whole day, the noise edges will be established by genotypes evaluated early in the day (broader noise segments), risking the loss of important spectral information from those assessed under lower relative humidity (usually after midday) and therefore possessing narrower noise segments.

Because of this, after the noise selection criteria (% and *N Size*) are established, the user has an opportunity to filter by considering all the measurements as one group (*Group*) or as individual scans (*Individual*). When the *group* filter is selected in a specific segment, the program analyzes each sample where the selection criteria are met, identifying the minor and major wavelengths that have problems in the spectral data file, and uses these two wavelengths to eliminate the noise from each sample uniformly. This is very similar to what is done visually, but with an objective approach. For the *individual* option, each sample will be filtered independently from the others, rescuing important information for modeling, or the use of SRIs.

### Scan analysis

In this module, the user will be able to analyze, identify and correct inconsistencies between spectral signatures from the same scan or plot, a problem that is often unnoticed. In general, for simplicity or to dilute any errors generated while collecting the data, there is a tendency to average samples within the same scan, which most of the time is done without any deeper analysis. As mentioned before, this should not be a complication when the data analysis considers a few measurements, but in breeding programs this search would be time consuming.

There are several aspects influencing the homogeneity between samples within the same scan, especially if the measurements were performed under field conditions. Unnoticed modification of the measurement angle during plot screening is probably the main source of variability. In practical terms it is difficult to maintain the exact angle of measurement, even for a few seconds (hand steadiness of the operator, distractions, or fatigue); each sample is derived from several integrations, usually more than 10, so the chance of making a mistake is not uncommon. When a plot is screened, it can be performed by keeping the fiber aimed at a single point (lower variability and representation) or across several plants (higher representation but greater variability); when the second option is taken, the chances of integrating other materials into a single scan or sample (e.g., soil, weeds, or air) are increased, and also enhanced by changes in measurement angles. Other considerations such as the effect of the wind speed or turbulence on the measured surface would be detected.

The user needs to set up the *Maximum variation coefficient* accepted for the samples belonging to the same scan. The software will find the scans where the limit is exceeded, at any wavelength, and this will be reported in the *Scans with problems* section. The samples that need to be checked can be individually analyzed on the graphical window, where it is possible to visualize all the samples in a single graph, identifying (zooming in and out) and deleting those spectral signatures with problems.

It is important to mention that the samples selected with problems within a same scan, do not necessarily need to be modified. This decision will depend on the magnitude of the differences between samples and the number of wavelengths involved. In cases where the user decides to intervene in a scan, it is possible to select and delete one or more samples from the *Samples to delete* section.

### Outlier analysis

This third filter is designed for rapid identification of problems associated with inconsistencies within spectral data. When outlier data is found, it will be necessary to evaluate the permanence of these in the data matrix.

Because of the high number of genotypes and samples per scan, it is difficult to identify data points that do not follow the general trends. Field experience has proven that is common to find small clouds of data whose main source of error comes from the calibration process. For example, the sun's movement throughout the day requires calibrations to be performed every 10–15 min. Due to distractions or tiredness during long working hours, the calibration can be forgotten, generating differences in the sun's incidence angle and therefore variations in the reflectance readings. Another form of user error, although less common and related to specific devices, may occur if the user has left the mouse cursor on one of the calibration icons (optimization, dark current, or white reference), performing an unconscious and incomplete calibration with a random click and thus generating undetectable reading errors.

In this module, it is possible to integrate a visual analysis of the reflectance and the response variable data at the same time. The user has four graphs to explore outlier information, evaluating different SRIs, and traits. In this section, it is also possible to *Edit* each graph, selecting data that need to be removed from the data matrix.

For these actions, the software will average the samples per scan to generate each SRI. This is important because the user should check the *Noise Analysis* and *Scan Analysis* modules first.

### Collinearity analysis

Collinearity or multicollinearity is a problem in regression analysis where the predictor variables “X” are themselves highly correlated (Draper and Smith, 2003). With the use of high-resolution spectral reflectance data, the collinearity problem is inherent to the data collection method employed because several wavelengths are highly correlated. If the goal is to understand how several predictor variables impact on a specific response variable “Y,” the collinearity is a big issue. Therefore, depending on the modeler's interest, it may be necessary to implement a collinearity analysis before construction of complex models (e.g., multilinear regression model).

In this module, the user can identify wavelengths that deliver the same predictive information for a given response variable, keeping only those that best explain it. This analysis can be performed (*collinearity test setting*) by *linear regression*, indicating the threshold coefficient of determination (*R square cutoff*), or through Artificial Neural Networks (ANN), considering a training process by Levenberg-Marquardt (trainlm), and Mean Squared Error (MSE) as a performance indicator. Depending on the data matrix and computer performance, the non-linear approach (ANN) could take several minutes or hours.

### Individual wavelength analysis

For the construction of new SRIs and regression models, it would be desirable to know the degree of dependency between individual wavelengths and the response variable. In this module, the researcher can study the behavior of each wavelength relative to each variable under study, considering one, or more of the following models:

1. Polynomial 1: *y* = *p*1 · *x* + *p*2
2. Polynomial 2: *y* = *p*1 · *x*^2^ + *p*2 · *x* + *p*3
3. Weibull: *y* = *p*1 · *p*2 · *x*^(*p*2−1)^ · *e*^(−*p*1 · *x*^*p*^2^^)^
4. Exponential: *y* = *p*1 · *e*^(*p*2 · *x*)^
5. Power: *y* = *p*1 + *p*2 · *x*^(*p*3)^
6. Logarithmic: *y* = *p*1 · ln (*x*) + *p*2

For this analysis, the user can select different statistics to sort the results (adjusted and non-adjusted determination coefficient, root mean squared error, sum of squares due to errors, and degree of freedom). It is also necessary to set up a minimum or maximum value for the selected statistics in order to export just those results (*Values above or below*). The exported file will show, for each wavelength, the statistics values for the selected model(s) where those minimum or maximum values were met.

This module and the following one (SRI analysis) work with sample averages, forcing the user to perform a deep preliminary analysis, thus avoiding any error in the data matrix.

### Spectral reflectance index (SRI) analysis

The implementation of concatenate formulas in spreadsheets is helpful for automating time-consuming procedures. However, due to the number of scans, samples per scans, measured wavelengths, evaluated response variables, and tested SRIs, the physical size of the resulting spreadsheets (several MB) implies the need for high performance computers.

By evaluating the same regression models reviewed with the previous function, the user will be able to identify the SRIs (initially 255: Jordan, 1969; Rouse et al., 1973; Rouse, 1974; Tucker, 1979; Hardisky et al., 1983; Guyot and Baret, 1988; Guyot et al., 1988; Huete, 1988; Baret et al., 1989; Clevers, 1989; Curran, 1989; Hunt and Rock, 1989; Major et al., 1990; Barnes et al., 1992, 2000; Chappelle et al., 1992; Gamon et al., 1992; Peñuelas et al., 1993a,b, 1994, 1995, 1997; Vogelmann et al., 1993; Carter, 1994; Gitelson and Merzlyak, 1994, 1997; McMurtrey et al., 1994; Qi et al., 1994; Roujean and Breon, 1995; Smith et al., 1995; Chen, 1996; Chen and Cihlar, 1996; Filella et al., 1996; Fourty et al., 1996; Gao, 1996; Ma et al., 1996; Rondeaux et al., 1996; Huete et al., 1997; van Deventer et al., 1997; Blackburn, 1998, 1999; Datt, 1998, 1999; Merton, 1998; Peñuelas and Filella, 1998; Gamon and Surfus, 1999; Gitelson et al., 1999, 2001, 2003, 2005, 2006; Merzlyak et al., 1999; Peñuelas and Inoue, 1999; Daughtry et al., 2000; Marshak et al., 2000; Thenkabail et al., 2000; Broge and Leblanc, 2001; Raun et al., 2001; Zarco-Tejada et al., 2001, 2003a,b, 2005; Broge and Mortensen, 2002; Haboudane et al., 2002, 2004; Read et al., 2002; Serrano et al., 2002; Sims and Gamon, 2002; Gupta et al., 2003; Hansen and Schjoerring, 2003; Steddom et al., 2003; Viña, 2003; Dash and Curran, 2004; Gandia et al., 2004; Le Maire et al., 2004, 2008; Schlemmer et al., 2005; Zhao et al., 2005; Vincini et al., 2006; Babar et al., 2006a,b; Mirik et al., 2006a,b; Inoue et al., 2007, 2008; Prasad et al., 2007; Rodríguez-Pérez et al., 2007; Zhu et al., 2007; Rama Rao et al., 2008; White et al., 2008; Wu et al., 2008a,b; Richter et al., 2009; Serbin et al., 2009; Stroppiana et al., 2009; Yañez et al., 2009; Dzikiti et al., 2010; Herrmann et al., 2010; Mistele and Schmidhalter, 2010; Yao et al., 2010, 2011; Garrity et al., 2011; Hernández-Clemente et al., 2011; Main et al., 2011; Pimstein et al., 2011; Tian et al., 2011, 2014; Winterhalter et al., 2011; Wang et al., 2011a,b) having the higher adjusted coefficients of determination (*Adj. RSquare values above*) in relation to a response variable. Internally, the software will select all combinations (regression model, SRI, and response variable) where the adjusted coefficient of determination was reached. The *Export data* option will generate a report that includes all the statistics analyzed in the previous function for the best-evaluated regression model and for each one (in the case that more than two were tested).

For publication purposes this module also includes an exportable *Detailed index report*, where it is possible to select specific SRIs and response variables. The report will include the SRI and variable values for each of the measurements, allowing the user to create XY graphs.

## Operational examples of SK-UTALCA

### Testing data sets

During the 2011/12 growing season, 386 genotypes of wheat (*Triticum spp*. L.) from different breeding programs (INIA-Chile, INIA-Uruguay and CIMMYT) were assessed under three water regimens (fully irrigated, mild water deficit and severe water deficit). This trial was established at Santa Rosa Experimental Station (36° 32′ S, 71° 55′ W; 217 m.a.s.l.), Regional Research Center INIA Quilamapu (Chillán, VIII Region, Chile), considering an alpha-lattice design (386 genotypes + 2 cvs. replicated seven times to assess field variability) and two replications.

Reflectance measurements were performed using a portable spectroradiometer (FieldSpec® 3 Jr, ASD Inc., Boulder, CO, USA) (350–2500 nm), between 12:00 and 16:00 h, on clear days (solar radiation higher than 800 Wm^−2^). Prior to the first measurement and every 15 min, the equipment was calibrated using a field reference panel (Spectralon, ASD Inc., Boulder, CO, USA). The equipment was configured to read three samples per scan. Each plot (genotype) was scanned once.

A detailed methodology can be found in Lobos et al. (2014). For purposes of this article, only one environment (fully irrigated), one phenological stage (grain filling) and one replicate will be considered.

### Data analysis

In this section, we highlight some of the key results of the analysis performed using the SK-UTALCA software.

#### Setting up

Prior to analysis the user needs to: (i) load the spectral data file (denoted as “*x*”); (ii) load the response variable(s) file (denoted as “*y*”); and (iii) define the number of samples per scan (in this case three). Wavelengths need to be placed in columns and samples in rows; the *transpose data* function is available.

**Figure 1:**
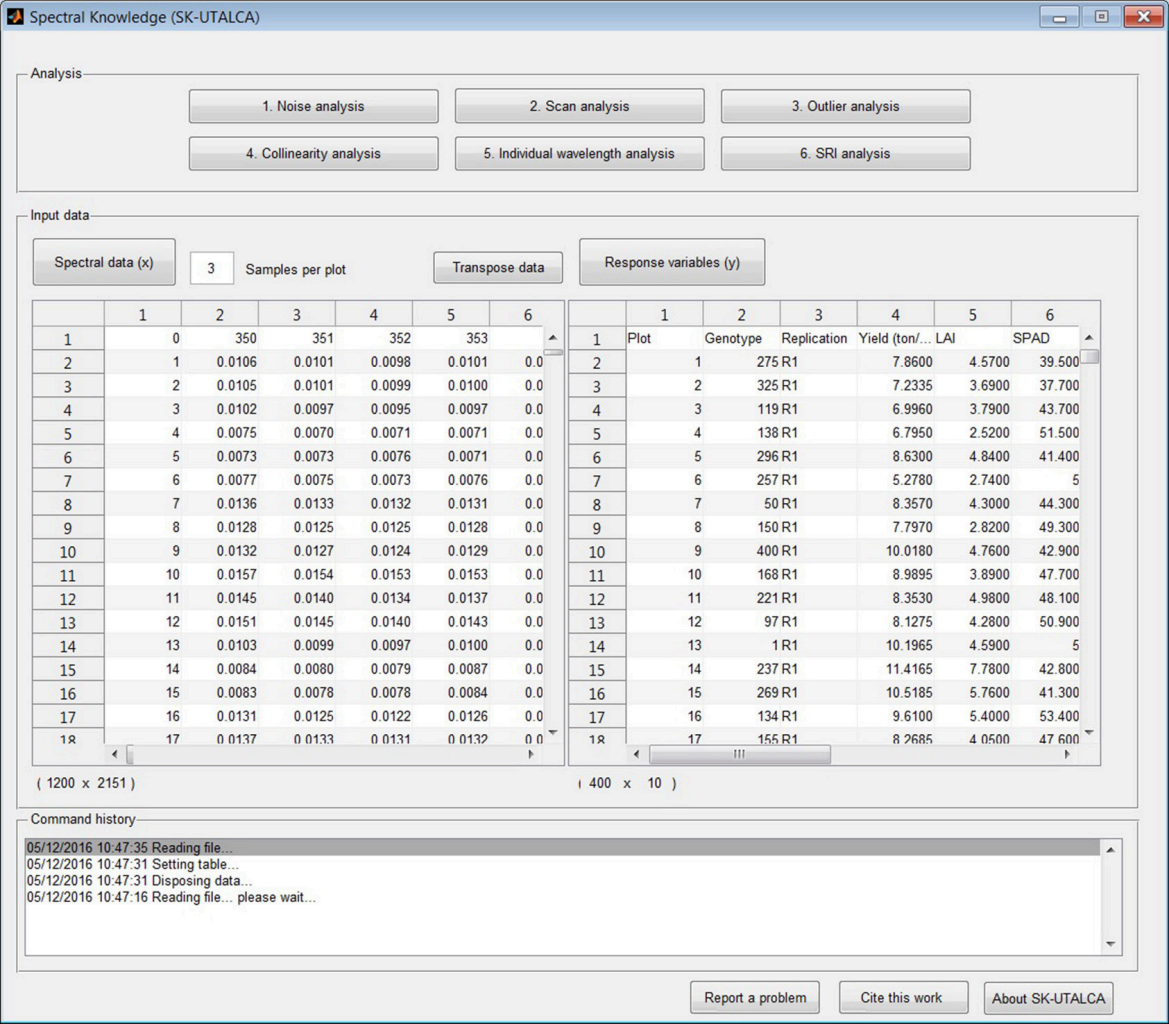
**Main screen divided horizontally into three sections: analysis, input data, and command history**. Screen shows loaded databases (spectral and response variable data files); the *transpose data* option is also available for the spectral matrix.

The file format for the spectra (*Genotype, Wavelength*_1_, *Wavelength*_2_, *Wavelength*_3_, …*Wavelength*_*n*_) and the response variables (*Plot, Genotype, Replication, Variable*_1_*, Variable*_2_*,…Variable*_*n*_) are presented in Figure 1. If for any reason the user realizes that there are missing plots (no spectral information) before the spectral data is uploaded, keeping in mind the sample number per scan, those rows can be left empty. If calibration data is among the spectral data output from the spectrometer, it should be removed prior to uploading the reflectance data (*x*).

Once the data has been loaded into the software and the wavelengths are arranged into columns, it is possible to start the analysis.

#### Noise analysis

**Figure 2:**
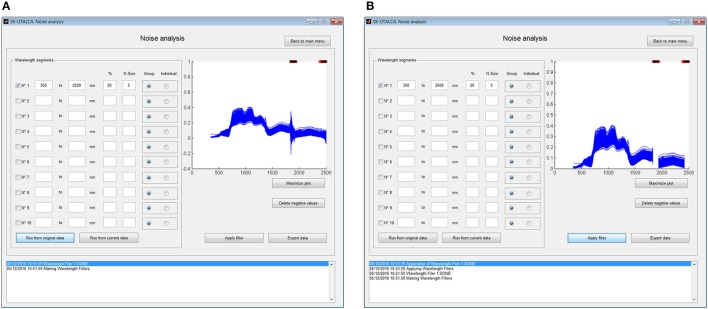
**Example of noise analysis showing 400 scans (x 3 samples ea.) prior to (A)** and after **(B)** the noise filter was applied. On both windows, red crosses (top) show where the maximum percentage of variations was exceeded and black crosses (top) where both criteria (% and the number of neighbors) were detected.

To apply this filter it is necessary to indicate the wavelength segment for analysis, the cutting criteria (*Group* or *Individual*), the maximum percentage of variations accepted (%), and the number of neighbors (*N size*). The selection of each wavelength segment and the criteria for each one (% and *N Size*) will depend on the user experience and the environmental conditions where the measurements were taken; for example, noise at 1800–1950 nm and 2350–2500 nm is usually wider and stronger than at 1300–1400 nm, so the criteria should consider higher values of % and *N Size* for the first two segments. In this operational example, the filter was applied to the whole spectral range (350–2500 nm) considering a group filter, with five wavelengths as *N size* and a maximum accepted variation among them of 20%. Figure 2 shows the results prior to (A) and after (B) the filter was applied. In this case, the filter was able to detect two main noise zones from 1833 to 1935 nm and from 2422 to 2500 nm.

#### Scan analysis

**Figure 3:**
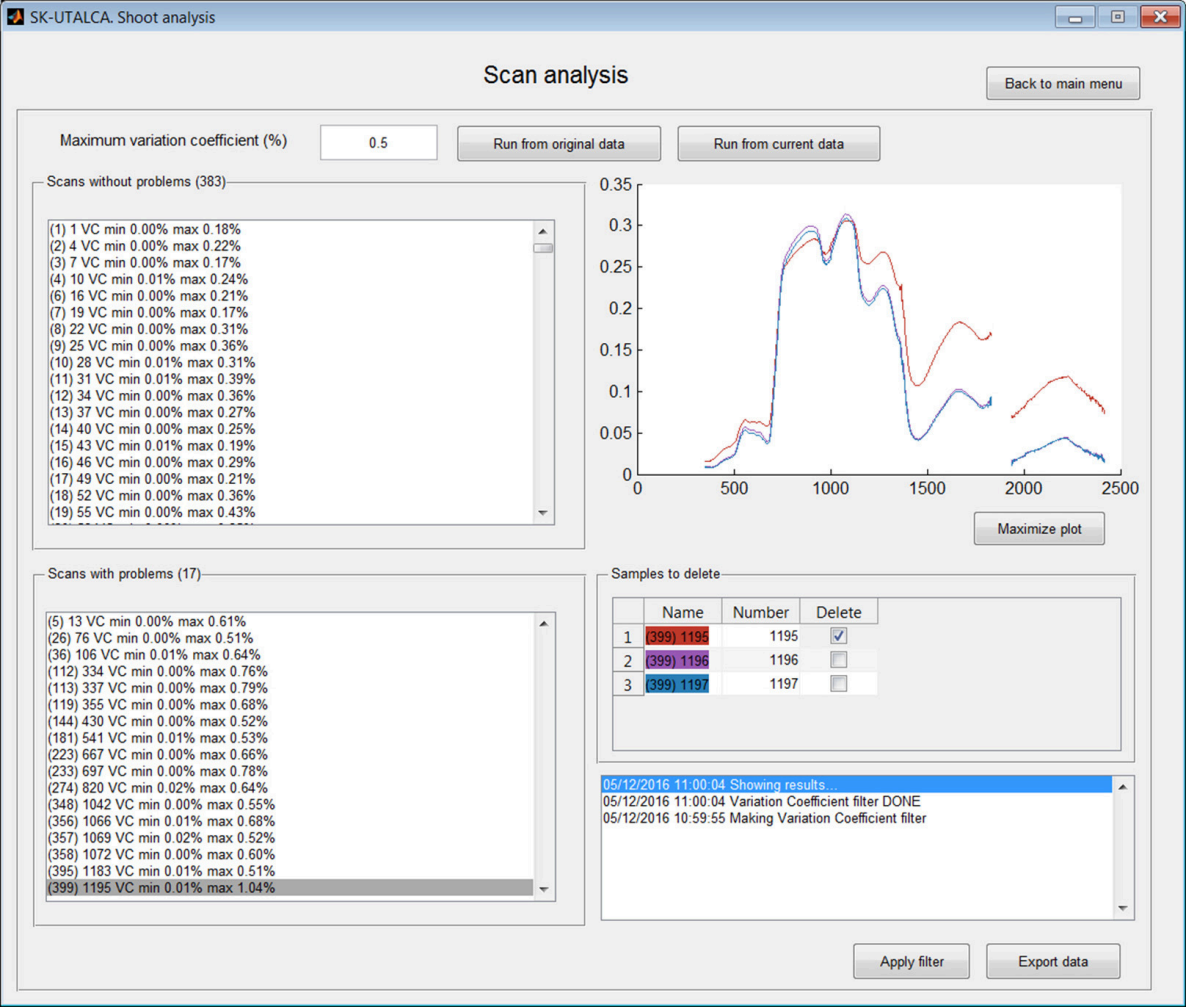
**Example of scan analysis**. The software divided the scans or plots between those that did not surpass the maximum accepted variation coefficient (*Scans without problems*) and those where it was exceeded (*Scans with problems*). Scan 399 was selected, and its first sample (red) was identified for deletion (*Apply filter*).

The Scan analysis module allows detection of abnormal variations among samples within the same scan. In this operational example, the Scan analysis was applied using the function *Run from the current data*, that is to say, considering the results obtained using previous filter (without spectral noise). The *Maximum variation coefficient* was set at 0.5%. The software was able to select 383 scans or plots without problems and 17 where the threshold was exceeded (5, 26, 36, 112–113, 119, 144, 181, 223, 233, 274, 348, 356–358, 395, and 399). In the Figure 3, scan or plot 399 is graphed, and the first sample (1195 on red) was selected for deletion. This result could be an indicator of a measurement problem associated with the operator (modification of the measurement angle) or external conditions (e.g., wind speed) during the first sample integrations. In case of all samples from a specific scan need to be deleted, the software will maintain this scan as empty rows, avoiding problems in further analyses.

#### Outlier analysis

**Figure 4:**
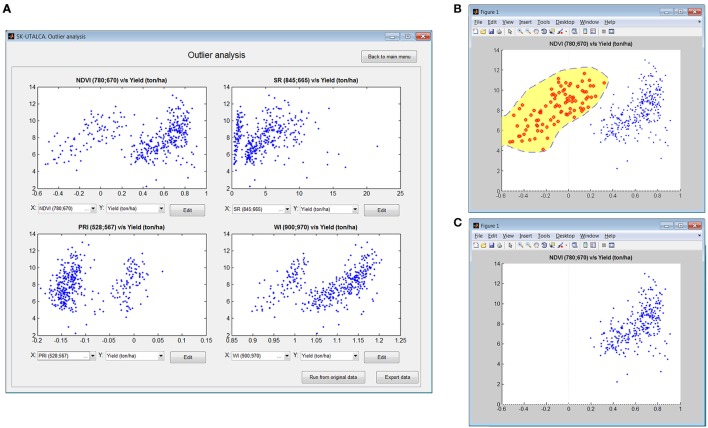
**Example of outlier analysis showing four scatterplot graphs (NDVI, SR, PRI and WI vs. Yield) (A)**. Using the *Edit* function, NDVI vs. Yield was used to select scans with NDVI values below 0.31 **(B)** for deletion **(C)**.

This module is a simple and exploratory analysis to identify outlier scans, allowing the user to detect field measurement problems (e.g., calibration). Four scatterplot graphs will show the relationship between any SRI available on the software database and the loaded response variables. If a problem is detected, it is possible to use the *Edit* option to manually remove the samples. In this operational example, the relationship between NDVI and Yield was used to inspect the possible outlier samples. Figure 4A shows how different SRIs (NDVI, SR, PRI, and WI) can generate different data distributions, helping the user in cases where problems are not so evident; on the top left graph (NDVI vs. Yield) two clouds of data points can be identified, divided at the NDVI value of 0.31.

Once the information from the smaller data cloud was analyzed (NDVI < 0.31), it was evident that the data set corresponded to 96 contiguous scans or plots (104–200), suggesting that there were problems associated with the measurement. When information from the spectrometer was checked, it was concluded that the operator had skipped one calibration. It is always important to check the pertinence of negative SRI values because they are probably related to measurement errors.

After identification of the origin of a particular problem, any graph can be selected for editing. In this example (NDVI vs. Yield), the scan with the problem can be selected (Figure 4B) and deleted (Figure 4C).

#### Collinearity analysis

**Figure 5:**
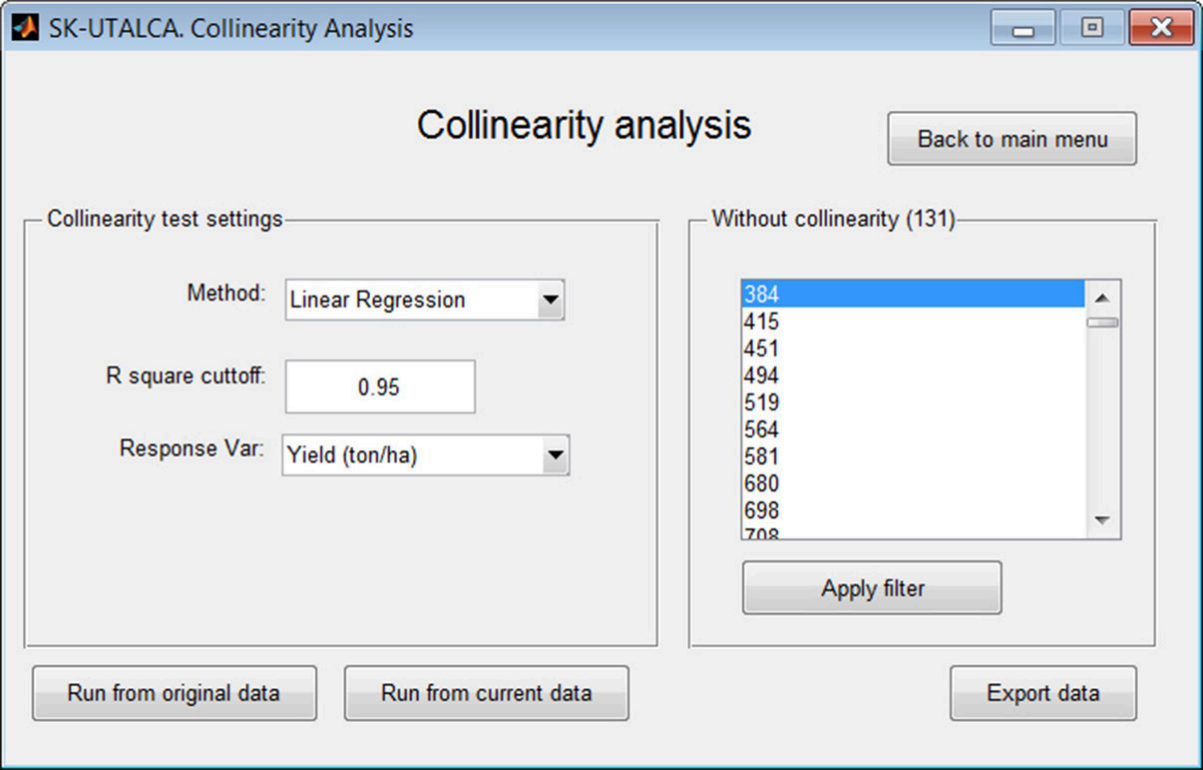
**Example of collinearity analysis for deletion of wavelengths delivering the same predictive information for Yield**. The analysis, considering a linear regression method (*R square cutoff* = 0.95), selected 131 wavelengths without collinearity.

Using linear regression or ANN, the collinearity analysis module identifies wavelengths that delivering the same predictive information for a given response variable, keeping only those that best explain it. In this operational example, collinearity analysis was applied by considering the results obtained from the scan analysis (*Run from current data*), with Yield being the response variable in the linear regression (*R square cutoff* = 0.95). Results of this analysis found 131 wavelengths without collinearity (Figure 5).

#### Individual wavelength analysis

**Figure 6:**
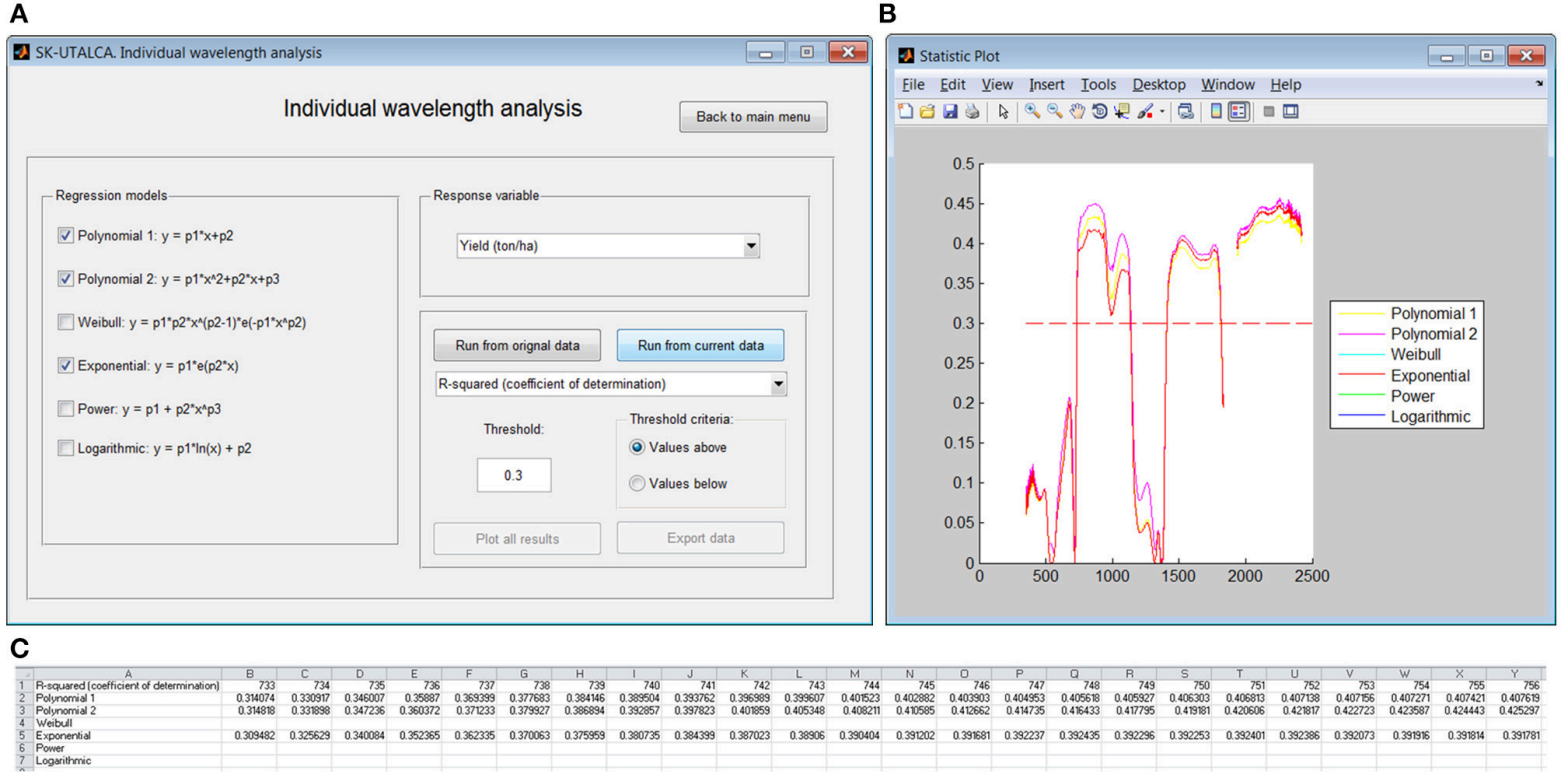
**Example of the individual wavelength analysis module**. The relationships were searched by considering Yield and a coefficient of determination higher than 0.3 **(A)**. Results were plotted for visual analysis **(B)** and exported to a spreadsheet **(C)**.

In this module it is possible to assess the relationship of individual wavelengths and a given response variable. Three regression models were selected (*Polynomial* 1 and *2*, and *Exponential*) to search for wavelengths with determination coefficients *above* 0.3 in relation to Yield (Figure 6A). If the user selects *Plot all results*, a graph will show the wavelengths below and above the determination coefficient cutoff (Figure 6B). These results can be exported to a spreadsheet; for each selected regression model, only wavelengths where the chosen statistic surpassed the cutoff will be shown (Figure 6C). In this operational example, there were three groups of wavelengths with determination coefficients above the threshold: 733–1139, 1409–1815, and 1936–2421 nm.

#### Spectral reflectance index (SRI) analysis

**Figure 7:**
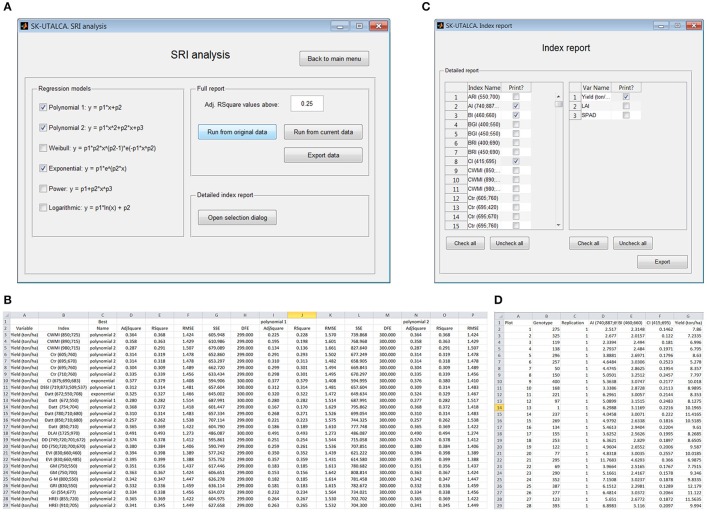
**Example of the SRI analysis module**. Three regression models were selected to search for SRI and response variables with a minimum adjusted coefficient of determination of 0.25 **(A)**. The exported file shows the adjusted coefficient of determination for the best approximation (*Best*) and for each selected regression model **(B)**. When a detailed report is required **(C)**, the SRI value for each scan is calculated automatically **(D)**.

As in the previous module, when different regression models were considered, SRI analysis has the option to evaluate the relationship between all loaded response variables and all SRIs available in the software database. For this example, three regression models were selected (*Polynomial* 1 and *2*, and *Exponential*) to search the SRIs and response variables with an adjusted determination coefficient higher than 0.25 (Figure 7A). When *Export data* is selected, all relationships with adjusted determination coefficients higher than 0.25 will be reported (Figure 7B); the results, which are organized according to the loaded variables (column A) and SRIs (column B), show which regression model had the highest determination coefficient (*Best*) for each SRI, as well as its statistics [adjusted and non-adjusted determination coefficient, root mean squared error (RMSE), sum of squares due to errors (SSE) and degree of freedom (DFE)] (columns C–H). The results for each evaluated regression model are also described (*Polynomial 1*: columns I–M; *Polynomial 2*: columns N–R, and so on). In this screen example, the adjusted *R*^2^ varied between 0.257 (Datt 850;710;680) and 0.406 (DLAI 1725;970), with these SRIs having the highest and lowest RMSEs, respectively (Figure 7B).

The selection of *Open selection dialog* (*Detailed index report*) enables the user to select specific SRIs and response variables for figure elaboration (*Index report*, Figure 7C). In this operational example, three SRIs (AI, BI, and CI) and one response variable (Yield) were selected. The SRI value for each scan or plot is given (Figure 7D) so the user can generate XY scatter plots for each tested SRI (X) and response variable (Y).

## Conclusions

Spectral Knowledge (SK-UTALCA) is a software package that allows an easy and fast exploratory analysis of high-resolution spectral reflectance data, providing the user with tools to detect measurement problems and the generation of key information for later modeling. SK-UTALCA is especially useful for plant breeding or any other research area where the number of measurements (big data files) involves long working hours that increase the risk of making involuntarily mistakes. This freely-available software is the result of several years of measurements and analysis of spectral data oriented toward the prediction of traits in plant breeding.

## Author contributions

GL, CP-E contributed equally toward the intellectual input into the final version of this paper, including development of the software, data analysis, interpreting, and discussion of the results, and writing and editing the manuscript.

### Conflict of interest statement

The authors declare that the research was conducted in the absence of any commercial or financial relationships that could be construed as a potential conflict of interest.

## Supplementary material

### Software availability

The compiled version of the software will be available for free downloading at http://www.fenomica.utalca.cl/ and source code is available as Supplementary Material.
