# Supplementary material for: Spectral Knowledge (SK-UTALCA): Software for Exploratory Analysis of High-Resolution Spectral Reflectance Data on Plant Breeding
Source: Front Plant Sci. 2017 Jan 9;7:1996. doi: 10.3389/fpls.2016.01996 (PMC5220079; doi:10.3389/fpls.2016.01996)
Supplement: Supplementary file 1 [file Table1.DOCX]

Supplementary Table 1. Spectral Knowledge (SK-UTAL): available spectral reflectance indices.

| N° | Name | Index | Formula | Reference |
| --- | --- | --- | --- | --- |
| 1 | Anthocyanin Reflectance Index (550;700)p | ARI (550;700) | (1 / R550) - (1 / R700) | Gitelson et al., 2001 |
| 2 | Aphid Index (740;887;691;698) | AI (740;887;691;698) | (R740 - R887) / (R691 - R698) | Mirik et al., 2006a |
| 3 | BI (460;660) | BI (460;660) | 6 * (R460 / R660) | Inoue et al., 2008 |
| 4 | Blue Green Index (400;550) | BGI (400;550) | R400 / R550 | Zarco-Tejada et al., 2005 |
| 5 | Blue Green Index (450;550) | BGI (450;550) | R450 / R550 | Zarco-Tejada et al., 2005 |
| 6 | Blue Red Index (400;690) | BRI (400;690) | R400 / R690 | Zarco-Tejada et al., 2005 |
| 7 | Blue Red Index (450;690) | BRI (450;690) | R450 / R690 | Zarco-Tejada et al., 2005 |
| 8 | Canopy index (415;695) | CI (415;695) | R415 / R695 | Read et al., 2002 |
| 9 | Canopy Water Mass Index (850;725) | CWMI (850;725) | R850 / R725 | Winterhalter et al., 2011 |
| 10 | Canopy Water Mass Index (890;715) | CWMI (890;715) | R890 / R715 | Winterhalter et al., 2011 |
| 11 | Canopy Water Mass Index (980;715) | CWMI (980;715) | R980 / R715 | Winterhalter et al., 2011 |
| 12 | Carter (605;760) | Ctr (605;760) | R605 / R760 | Carter, 1994 |
| 13 | Carter (695;420) | Ctr (695;420) | R695 / R420 | Carter, 1994 |
| 14 | Carter (695;670) | Ctr (695;670) | R695 / R670 | Carter, 1994 |
| 15 | Carter (695;760) | Ctr (695;760) | R695 / R760 | Carter, 1994 |
| 16 | Carter (710;760) | Ctr (710;760) | R710 / R760 | Carter, 1994 |
| 17 | Curvature Index (675;690;683) | CI (675;690;683) | (R675 * R690) / (R683 * R683) | Zarco-Tejada et al., 2003a |
| 18 | Damage Sensitive Spectral Index (719;873;509;537) | DSSI (719;873;509;537) | (R719 - R873 - R509 - R537) / ((R719 - R873) + (R509 - R537)) | Mirik et al., 2006b |
| 19 | Damage Sensitive Spectral Index (747;901;537;572) | DSSI (747;901;537;572) | (R747 - R901 - R537 - R572) / ((R747 - R901) + (R537 - R572)) | Mirik et al., 2006b |
| 20 | Datt (672;550;708) | Datt (672;550;708) | R672 / (R550 * R708) | Datt, 1999 |
| 21 | Datt (672;550) | Datt (672;550) | R672 / R550 | Datt, 1998 |
| 22 | Datt (754;704) | Datt (754;704) | R754 / R704 | Datt, 1999 |
| 23 | Datt (780;710;680) | Datt (780;710;680) | (R780 - R710) / (R780 - R680) | Datt, 1999 |
| 24 | Datt (850;710;680) | Datt (850;710;680) | (R850 - R710) / (R850 - R680) | Datt, 1999 |
| 25 | Datt (850;710) | Datt (850;710) | R850 / R710 | Datt, 1999 |
| 26 | Datt (860;550;708) | Datt (860;550;708) | R860 / (R550 * R708) | Datt, 1998 |
| 27 | Difference LAI (1725;970) | DLAI (1725;970) | R1725 - R970 | Le Maire et al., 2008 |
| 28 | Double Difference Index (749;720;701;672) | DD (749;720;701;672) | (R749 - R720) - (R701 - R672) | Le Maire et al., 2004 |
| 29 | Double Difference Index (750;720;700;670) | DD (750;720;700;670) | (R750 - R720) - (R700 - R670) | Le Maire et al., 2004 |
| 30 | Enhanced Vegetation Index (830;660;460) | EVI (830;660;460) | 2.5 * (R830 - R660) / (1 + R830 + 6 * R660 - 7.5 * R460 ) | Huete et al., 1997 |
| 31 | Enhanced Vegetation Index (830;660;485) | EVI (830;660;485) | 2.5 * (R830 - R660) / (1 + R830 + 6 * R660 - 7.5 * R485 ) | Huete et al., 1997 |
| 32 | Gitelson and Merzlyak (750;550) | GM (750;550) | R750 / R550 | Gitelson and Merzlyak, 1997 |
| 33 | Gitelson and Merzlyak (750;700) | GM (750;700) | R750 / R700 | Gitelson and Merzlyak, 1997 |
| 34 | Green Model Index (800;550) | G-M (800;550) | (R800 / R550) - 1 | Gitelson et al., 2005 |
| 35 | Green Ratio Index (830;550) | GRI (830;550) | R830 / R550 | Inoue et al., 2008 |
| 36 | Greenness Index (554;677) | GI (554;677) | R554 / R677 | Smith et al., 1995 |
| 37 | Hyperspectral Red Edge Index (855;720) | HREI (855;720) | (R855 - R720) / (R855 + R720) | Thenkabail et al., 2014 |
| 38 | Hyperspectral Red Edge Index (910;705) | HREI (910;705) | (R910 - R705) / (R910 + R705) | Thenkabail et al., 2014 |
| 39 | Hyperspectral Water and Moisture Index (855;970) | HWMI (855;970) | (R855 - R970) / (R855 + R970) | Thenkabail et al., 2014 |
| 40 | MCARI / OSAVI (750;705;550) | MCARI / OSAVI (750;705;550) | ((R750 - R705) - 0.2 * (R750 - R550)) * (R750 / R705) / (1 + 0.16) * (R750 - R705) / (R750 + R705 + 0.16) | Wu et al., 2008b |
| 41 | MERIS Terrestrial Chlorophyll Index (750;710;680) | MTCI (750;710;680) | (R750 - R710) / (R710 - R680) | Dash and Curran, 2004 |
| 42 | MERIS Terrestrial Chlorophyll Index (754;709;681) | MTCI (754;709;681) | (R754 - R709) / (R709 - R681) | Dash and Curran, 2004 |
| 43 | MERIS Terrestrial Chlorophyll Index (800;750;670) | MTCI (800;750;670) | (R800 - R750) / (R750 - R670) | Dash and Curran, 2004 |
| 44 | Modified Carotenoid Concentration Index (510;550;780) | mCRI (510;550;780) | ((1 / R510) - (1 / R550)) * R780 | Gitelson et al., 2006 |
| 45 | Modified Carotenoid Concentration Index (510;700;770) | mCRI (510;700;770) | ((1 / R510) - (1 / R700)) * R770 | Gitelson et al., 2003 |
| 46 | Modified Chlorophyll Absorption in Reflectance Index (700; 670;550) | MCARI (700; 670;550) | ((R700 - R670) - (0.2 * (R700 - R550))) * (R700 / R670) | Daughtry et al., 2000 |
| 47 | Modified Chlorophyll Absorption in Reflectance Index (750;705;550) | MCARI (750;705;550) | ((R750 - R705) - (0.2 * (R750 - R550))) * (R750 / R705) | Wu et al., 2008b |
| 48 | Modified Chlorophyll Absorption in Reflectance Index (800;670;550) | MCARI (800;670;550) | 1.5 * ( 2.5 * (R800 - R670) - 1.3 * (R800 - R550)) / √((2 * R800 + 1) * 2 - (6 * R800 - 5* √(R670)) - 0.5) | Haboudane et al., 2004 |
| 49 | Modified Red Edge Normalized Difference Vegetation Index (750;705;445) | mNDVI (750;705;445) | (R750 - R705) / (R750 + R705- 2 * R445) | Sims and Gamon, 2002 |
| 50 | Modified Red Edge Normalized Difference Vegetation Index (800;680;445) | mNDVI (800;680;445) | (R800 - R680) / (R800 + R680 - 2 * R445) | Sims and Gamon, 2002 |
| 51 | Modified Simple Ratio (600;680) | mSR (600;680) | ((R600 / R680) - 1) / (√(R600 / R680) + 1) | Chen, 1996 |
| 52 | Modified Simple Ratio (750;705) | mSR (750;705) | ((R750 / R705) - 1) / (√(R750 / R705) + 1) | Chen, 1996 |
| 53 | Modified Simple Ratio (800;445;680) | mSR (800;445;680) | (R800 - R445) / (R680 - R445) | Sims and Gamon, 2002 |
| 54 | Modified Simple Ratio (845;665) | mSR (845;665) | ((R845 / R665) - 1) / (√(R845 / R665) + 1) | Chen, 1996 |
| 55 | Modified Simple Red Edge Ratio Index (750;445;705) | mSR (750;445;705) | (R750 - R445) / (R705 - R445) | Sims and Gamon, 2002 |
| 56 | Modified Soil Adjusted Vegetation Index (800;670) | MSAVI (800;670) | (0.5 * (2 * R800 + 1 - √((2 * R800 + 1) * (2 * R800 + 1)))) - (8 * (R800 - R670)) | Qi et al., 1994 |
| 57 | Modified Soil Adjusted Vegetation Index (830;660) | MSAVI (830;660) | (1 + 0.5) * (R830 - R660) / (R830 + R660 + 0.5) | Inoue et al., 2008 |
| 58 | Modified Triangular Vegetation Index (800;550;670) | MTVI (800;550;670) | 1.2 * ( 1.2 * (R800 - R550) - 2.5 * (R670 - R550)) | Haboudane et al., 2004 |
| 59 | Modified Triangular Vegetation Index (880;554;758) | MTVI (880;554;758) | 1.2 * ( 1.2 * (R880 - R554) - 2.5 * (R758 - R554)) | Rodríguez-Pérez et al., 2007 |
| 60 | Modified Vegetation Stress Ratio (723;700) | MVSR (723;700) | R723 / R700 | White et al., 2008 |
| 61 | Moister Stress Index (1650;835) | MSI (1650;835) | R1650 / R835 | Hunt and Rock, 1989 |
| 62 | Moister Stress Index (1662;927) | MSI (1662;927) | R1662 / R927 | Thenkabail et al., 2000 |
| 63 | Moister Stress Index (870;1350) | MSI (870;1350) | R870 / R1350 | Rodríguez-Pérez et al., 2007 |
| 64 | Moisture Stress Index (1650;850) | MSI (1650;850) | R1650 / R850 | Hunt and Rock, 1989 |
| 65 | Narrow Band Normalized Difference Vegetation Index (850;680) | NBNDVI (850;680) | (R850 - R680) / (R850 + R680) | Thenkabail et al., 2000 |
| 66 | Nitrogen Reflectance Index (1510;660) | NRI (1510;660) | (R1510 - R660) / (R1510 + R660) | Herrmann et al., 2010 |
| 67 | Nitrogen Reflectance Index (570;670) | NRI (570;670) | (R570 - R670) / (R570 + R670) | Filella et al., 1995 |
| 68 | Normalized Difference Chlorophyll Index (762;527) | NDCI (762;527) | (R762 - R527) / (R762 + R527) | Marshak et al., 2000 |
| 69 | Normalized Difference Green Blue (573;440) | NDg-b (573;440) | (R573 - R440) / (R573 + R440) | Hansen and Schjoerring, 2003 |
| 70 | Normalized Difference Infrared Index (835;1650) | NDII (835;1650) | (R835 - R1650) / (R835 + R1650) | Hardisky et al., 1983 |
| 71 | Normalized Difference Infrared Index (860;1650) | NDII (860;1650) | (R860 - R1650) / (R860 + R1650) | Hardisky et al., 1983 |
| 72 | Normalized Difference Lignin Index (1754;1680) | NDLI (1754;1680) | (log(1 / R1754) - log(1 / R1680)) / (log(1 / R1754) + log(1 / R1680)) | Serrano et al., 2002 |
| 73 | Normalized Difference Moisture Index (1649;1722) | NDMI (1649;1722) | (R1649 - R1722) / (R1649 + R1722) | Wang et al., 2011a |
| 74 | Normalized Difference Moisture Index (1650;850) | NDMI (1650;850) | (R1650 - R850) / (R1650 + R850) | Inoue et al., 2008 |
| 75 | Normalized Difference Moisture Index (2200;1100) | NDMI (2200;1100) | (R2200 - R1100) / (R2200 + R1100) | Inoue et al., 2008 |
| 76 | Normalized Difference Nitrogen Index (1754;1680) | NDNI (1510;1680) | (log(1 / R1510) - log(1 / R1680)) / (log(1 / R1510) + log(1 / R1680)) | Fourty et al., 1996 |
| 77 | Normalized Difference Red Edge Index (790;720) | NDRE (790;720) | (R790 - R720) / (R790 + R720) | Barnes et al., 2000 |
| 78 | Normalized Difference Spectral Index (1050;1122) | NDSI (1050;1122) | (R1050 - R1122) / (R1050 + R1122) | Inoue et al., 2008 |
| 79 | Normalized Difference Spectral Index (1053;1058) | NDSI (1053;1058) | (R1053 - R1058) / (R1053 + R1058) | Inoue et al., 2008 |
| 80 | Normalized Difference Spectral Index (1060;1118) | NDSI (1060;1118) | (R1060 - R1118) / (R1060 + R1118) | Inoue et al., 2008 |
| 81 | Normalized Difference Spectral Index (1107;1110) | NDSI (1107;1110) | (R1107 - R1110) / (R1107 + R1110) | Inoue et al., 2008 |
| 82 | Normalized Difference Spectral Index (1220;710) | NDSI (1220;710) | (R1220 - R710) / (R1220 + R710) | Zhu et al., 2007 |
| 83 | Normalized Difference Spectral Index (1650;830) | NDSI (1650;830) | (R1650 - R830) / (R1650 + R830) | Inoue et al., 2007 |
| 84 | Normalized Difference Spectral Index (2215;830) | NDSI (2215;830) | (R2215 - R830) / (R2215 + R830) | Inoue et al., 2007 |
| 85 | Normalized Difference Spectral Index (403;830) | NDSI (403;830) | (R403 - R830) / (R403 + R830) | Inoue et al., 2008 |
| 86 | Normalized Difference Spectral Index (410;550) | NDSI (410;550) | (R410 - R550) / (R410 + R550) | Inoue et al., 2008 |
| 87 | Normalized Difference Spectral Index (410;710) | NDSI (410;710) | (R410 - R710) / (R410 + R710) | Inoue et al., 2008 |
| 88 | Normalized Difference Spectral Index (413;416) | NDSI (413;416) | (R413 - R416) / (R413 + R416) | Inoue et al., 2008 |
| 89 | Normalized Difference Spectral Index (420;970) | NDSI (420;970) | (R420 - R970) / (R420 + R970) | Inoue et al., 2008 |
| 90 | Normalized Difference Spectral Index (422;406) | NDSI (422;406) | (R422 - R406) / (R422 + R406) | Inoue et al., 2008 |
| 91 | Normalized Difference Spectral Index (422;416) | NDSI (422;416) | (R422 - R416) / (R422 + R416) | Inoue et al., 2008 |
| 92 | Normalized Difference Spectral Index (422;419) | NDSI (422;419) | (R422 - R419) / (R422 + R419) | Inoue et al., 2008 |
| 93 | Normalized Difference Spectral Index (442;435) | NDSI (442;435) | (R442 - R435) / (R442 + R435) | Inoue et al., 2008 |
| 94 | Normalized Difference Spectral Index (442;438) | NDSI (442;438) | (R442 - R438) / (R442 + R438) | Inoue et al., 2008 |
| 95 | Normalized Difference Spectral Index (450;1330) | NDSI (450;1330) | (R450 - R1330) / (R450 + R1330) | Inoue et al., 2008 |
| 96 | Normalized Difference Spectral Index (503;483) | NDSI (503;483) | (R503 - R483) / (R503 + R483) | Stroppiana et al., 2009 |
| 97 | Normalized Difference Spectral Index (518;676) | NDSI (518;676) | (R518 - R676) / (R518 + R676) | Inoue et al., 2008 |
| 98 | Normalized Difference Spectral Index (520;710) | NDSI (520;710) | (R520 - R710) / (R520 + R710) | Inoue et al., 2008 |
| 99 | Normalized Difference Spectral Index (530;550) | NDSI (530;550) | (R530 - R550) / (R530 + R550) | Inoue et al., 2008 |
| 100 | Normalized Difference Spectral Index (542;550) | NDSI (542;550) | (R542 - R550) / (R542 + R550) | Inoue et al., 2008 |
| 101 | Normalized Difference Spectral Index (543;548) | NDSI (543;548) | (R543 - R548) / (R543 + R548) | Inoue et al., 2008 |
| 102 | Normalized Difference Spectral Index (550;410) | NDSI (550;410) | (R550 - R410) / (R550 + R410) | Inoue et al., 2008 |
| 103 | Normalized Difference Spectral Index (620;623) | NDSI (620;623) | (R620 - R623) / (R620 + R623) | Inoue et al., 2008 |
| 104 | Normalized Difference Spectral Index (620;637) | NDSI (620;637) | (R620 - R637) / (R620 + R637) | Inoue et al., 2008 |
| 105 | Normalized Difference Spectral Index (682;553) | NDSI (682;553) | (R682 - R553) / (R682 + R553) | Gandia et al., 2004 |
| 106 | Normalized Difference Spectral Index (720;420) | NDSI (720;420) | (R720 - R420) / (R720 + R420) | Inoue et al., 2008 |
| 107 | Normalized Difference Spectral Index (750;761) | NDSI (750;761) | (R750 - R761) / (R750 + R761) | Inoue et al., 2008 |
| 108 | Normalized Difference Spectral Index (760;550) | NDSI (760;550) | (R760 - R550) / (R760 + R550) | Wang et al., 2011b |
| 109 | Normalized Difference Spectral Index (801;550) | NDSI (801;550) | (R801 - R550) / (R801 + R550) | Daughtry et al., 2000 |
| 110 | Normalized Difference Spectral Index (860;720) | NDSI (860;720) | (R860 - R720) / (R860 + R720) | Yao et al., 2010 |
| 111 | Normalized Difference Spectral Index (870;1450) | NDSI (870;1450) | (R870 - R1450) / (R870 + R1450) | Pimstein et al., 2011 |
| 112 | Normalized Difference Spectral Index (933;940) | NDSI (933;940) | (R933 - R940) / (R933 + R940) | Inoue et al., 2008 |
| 113 | Normalized Difference Spectral Index (933;948) | NDSI (933;948) | (R933 - R948) / (R933 + R948) | Inoue et al., 2008 |
| 114 | Normalized Difference Spectral Index (940;1122) | NDSI (940;1122) | (R940 - R1122) / (R940 + R1122) | Inoue et al., 2008 |
| 115 | Normalized Difference Spectral Index (962;964) | NDSI (962;964) | (R962 - R964) / (R962 + R964) | Inoue et al., 2008 |
| 116 | Normalized Difference Spectral Index (971;973) | NDSI (971;973) | (R971 - R973) / (R971 + R973) | Inoue et al., 2008 |
| 117 | Normalized Difference Spectral Index(565;533) | NDSI (565;533) | (R565 - R533) / (R565 + R533) | Tian et al., 2011 |
| 118 | Normalized Difference Tillage Index (1650;2215) | NDTI (1650;2215) | (R1650 - R2215) / (R1650 + R2215) | van Deventer et al., 1997 |
| 119 | Normalized Difference Vegetation Index (750;705) | NDVI (750;705) | (R750 - R705) / (R750 + R705) | Gitelson and Merzlyak, 1994 |
| 120 | Normalized Difference Vegetation Index (755;664) | NDVI (755;664) | (R755 - R664) / (R755 + R664) | Rouse, 1974 |
| 121 | Normalized Difference Vegetation Index (760;660) | NDVI (760;660) | (R760 - R660) / (R760 + R660) | Rouse et al., 1973 |
| 122 | Normalized Difference Vegetation Index (760;708) | NDVI (760;708) | (R760 - R708) / (R760 + R708) | Steddom et al., 2003 |
| 123 | Normalized Difference Vegetation Index (780;670) | NDVI (780;670) | (R780 - R670) / (R780 + R670) | Raun et al., 2001 |
| 124 | Normalized Difference Vegetation Index (800;600) | NDVI (800;600) | (R800 - R600) / (R800 + R600) | Ma et al., 1996 |
| 125 | Normalized Difference Vegetation Index (800;670) | NDVI (800;670) | (R800 - R670) / (R800 + R670) | Tucker, 1979 |
| 126 | Normalized Difference Vegetation Index (800;680) | NDVI (800;680) | (R800 - R680) / (R800 + R680) | Peñuelas et al., 1997 |
| 127 | Normalized Difference Vegetation Index (801;670) | NDVI (801;670) | (R801 - R670) / (R801 + R670) | Daughtry et al., 2000 |
| 128 | Normalized Difference Vegetation Index (807;736) | NDVI (807;736) | (R807 - R736) / (R807 + R736) | Yao et al., 2011 |
| 129 | Normalized Difference Vegetation Index (830;660) | NDVI (830;660) | (R830 - R660) / (R830 + R660) | Tucker, 1979 |
| 130 | Normalized Difference Vegetation Index (845;665) | NDVI (845;665) | (R845 - R665) / (R845 + R665) | Rouse, 1974 |
| 131 | Normalized Difference Vegetation Index (870;673) | NDVI (870;673) | (R870 - R673) / (R870 + R673) | Rodríguez-Pérez et al., 2007 |
| 132 | Normalized Difference Vegetation Index (880;673) | NDVI (880;673) | (R880 - R673) / (R880 + R673) | Zhao et al., 2005 |
| 133 | Normalized Difference Vegetation Index (884;680) | NDVI (884;680) | (R884 - R680) / (R884 + R680) | Rodríguez-Pérez et al., 2007 |
| 134 | Normalized Difference Vegetation Index (895;675) | NDVI (895;675) | (R895 - R675) / (R895 + R675) | Peñuelas et al., 1997 |
| 135 | Normalized Difference Vegetation Index (900;680) | NDVI (900;680) | (R900 - R680) / (R900 + R680) | Peñuelas et al., 1993b |
| 136 | Normalized Difference Vegetation Index (927;687) | NDVI (927;687) | (R927 - R687) / (R927 + R687) | Thenkabail et al., 2000 |
| 137 | Normalized Difference Water Index (860;1240) | NDWI (860;1240) | (R860 - R1240) / (R860 + R1240) | Gao, 1996 |
| 138 | Normalized Difference Water Index (870;1260) | NDWI (870;1260) | (R870 - R1260) / (R870 + R1260) | Rodríguez-Pérez et al., 2007 |
| 139 | Normalized Difference Water Index (970;850) | NDWI (970;850) | (R970 - R850) / (R970 + R850) | Babar et al., 2006b |
| 140 | Normalized Difference Water Index (970;880) | NDWI (970;880) | (R970 - R880) / (R970 + R880) | Prasad et al., 2007 |
| 141 | Normalized Difference Water Index (970;900) | NDWI (970;900) | (R970 - R900) / (R970 + R900) | Babar et al., 2006b |
| 142 | Normalized Difference Water Index (970;920) | NDWI (970;920) | (R970 - R920) / (R970 + R920) | Prasad et al., 2007 |
| 143 | Normalized Green Red Ratio 1 (673;554) | NGRR 1 (673;554) | (R673 - R554) / (R673 + R554) | Rodríguez-Pérez et al., 2007 |
| 144 | Normalized Green Red Ratio 2 (673;554) | NGRR 2 (673;554) | (R673 + R554) / (R673 - R554) | Rodríguez-Pérez et al., 2007 |
| 145 | Normalized Phaeophytinization Index (415;435) | NPQI (415;435) | (R415 - R435) / (R415 + R435) | Barnes et al., 1992 |
| 146 | Normalized Pigments Chlorophyll Index (680;430) | NPCI (680;430) | (R680 - R430) / (R680 + R430) | Peñuelas et al., 1994 |
| 147 | Optimized Soil Adjusted Vegetation Index (750;705) | OSAVI (750;705) | (1 + 0.16) * (R750 - R705) / (R750 + R705 + 0.16) | Main et al., 2011 |
| 148 | Optimized Soil Adjusted Vegetation Index (800;670) | OSAVI (800;670) | (1 + 0.16) * (R800 - R670) / (R800 + R670 + 0.16) | Rondeaux et al., 1996 |
| 149 | Orange Red Chlorophyll Absorption Ratio (630;680) | OCAR (630;680) | R630 / R680 | Schlemmer et al., 2005 |
| 150 | Photochemical Reflectance Index (512;531) | PRI (512;531) | (R512 - R531) / (R512 + R531) | Hernández-Clemente et al., 2011 |
| 151 | Photochemical Reflectance Index (528;567) | PRI (528;567) | (R528 - R567) / (R528 + R567) | Gamon et al., 1992 |
| 152 | Photochemical Reflectance Index (550;531) | PRI (550;531) | (R550 - R531) / (R550 + R531) | Gamon et al., 1992 |
| 153 | Photochemical Reflectance Index (570;530) | PRI (570;530) | (R570 - R530) / (R570 + R530) | Gamon et al., 1992 |
| 154 | Photochemical Reflectance Index (570;531;670) | PRI (570;531;670) | (R570 - R531 - R670) / (R570 + R531 + R670) | Hernández-Clemente et al., 2011 |
| 155 | Photochemical Reflectance Index (570;531) | PRI (570;531) | (R570 - R531) / (R570 + R531) | Gamon et al., 1992 |
| 156 | Photochemical Reflectance Index (570;539) | PRI (570;539) | (R570 - R539) / (R570 + R539) | Gamon et al., 1992 |
| 157 | Photochemical Reflectance Index * Chlorophyll Index (570;530; 760;700) | PRI * CI (570;530; 760;700) | (R570 - R530) / ((R570 + R530) * (R760 / (R700 - 1))) | Garrity et al., 2011 |
| 158 | Pigment Specific Normalised Difference (800;470) | PSND (800;470) | (R800 - R470) / (R800 + R470) | Blackburn, 1998 |
| 159 | Pigment Specific Normalised Difference (800;635) | PSND (800;635) | (R800 - R635) / (R800 + R635) | Blackburn, 1998 |
| 160 | Pigment Specific Normalised Difference (800;650) | PSND (800;650) | (R800 - R650) / (R800 + R650) | Blackburn, 1998 |
| 161 | Pigment Specific Normalised Difference (800;675) | PSND (800;675) | (R800 - R675) / (R800 + R675) | Blackburn, 1998 |
| 162 | Pigment Specific Simple Ratio (Cholophyll *a*) (800;680) | PSSRa (800;680) | R800 / R680 | Blackburn, 1998 |
| 163 | Pigment Specific Simple Ratio (Cholophyll *b*) (800;635) | PSSRb (800;635) | R800 / R635 | Blackburn, 1998 |
| 164 | Pigment Specific Simple Ratio C2 (800; 470) | PSSRc2 (800; 470) | R800 / R470 | Blackburn, 1998 |
| 165 | Pigment Specific Simple Ratio Chlorophyll *a* (810;676) | PSSRchla (810;676) | R810 / R676 | Blackburn, 1999 |
| 166 | PK Index (1645;1715) | PKI (1645;1715) | (R1645 - R1715) / (R1645 + R1715) | Pimstein et al., 2011 |
| 167 | Plant Biochemical Index (810;560) | PBI (810;560) | R810 / R560 | Rama Rao et al., 2008 |
| 168 | Plant Senescence Reflectance Index (678;500;750) | PSRI (678;500;750) | (R678 - R500) / R750 | Merzlyak et al., 1999 |
| 169 | Plant Senescence Reflectance Index (680;500;750) | PSRI (680;500;750) | (R680 - R500) / R750 | Merzlyak et al., 1999 |
| 170 | Ratio Analysis of Reflectance Spectra (746;513) | RARS (746;513) | R746 / R513 | Chappelle et al., 1992 |
| 171 | Ratio Analysis of Reflectance Spectra (Car) (760;500) | RARSc (760;500) | R760 / R500 | Chappelle et al., 1992 |
| 172 | Ratio Analysis of Reflectance Spectra (Chl *a*) (675;700) | RARSa (675;700) | R675 / R700 | Chappelle et al., 1992 |
| 173 | Ratio Analysis of Reflectance Spectra (Chl *b*) (675;650;700) | RARSb (675;650;700) | R675 / (R650 * R700) | Chappelle et al., 1992 |
| 174 | Ratio Index 1-dB (735;720) | RI-1 dB (735;720) | R735 / R720 | Gupta et al., 2003 |
| 175 | Ratio Index 2-dB (738;720) | RI-2 dB (738;720) | R738 / R720 | Gupta et al., 2003 |
| 176 | Ratio Index 3-dB (741;717) | RI-3 dB (741;717) | R741 / R717 | Gupta et al., 2003 |
| 177 | Ratio Index Half Power Point (747;708) | RI-half (747;708) | R747 / R708 | Gupta et al., 2003 |
| 178 | Ratio Vegetation Index (800;673) | RVI (800;673) | R800 / R673 | Broge and Mortensen, 2002 |
| 179 | Reciprocal of Moisture Stress Index (860;1650) | RMSI (860;1650) | R860 / R1650 | Hunt and Rock, 1989 |
| 180 | Red Blue Index (695;445) | RBI (695;445) | R695 / R445 | Rodríguez-Pérez et al., 2007 |
| 181 | Red Edge (670;780) | RE (670;780) | (R670 + R780) / 2 | Guyot and Baret, 1988 |
| 182 | Red Edge Inflection Point (670;780;700;740) | REIP (670;780;700;740) | (700 + 40 * ((R670 + R780) / (2 - R700))) / (R740 - R700) | Guyot et al., 1988 |
| 183 | Red Edge Model Index (750;720) | R-M (750;720) | (R750 / R720) - 1 | Gitelson et al., 2005 |
| 184 | Red Edge Reflectance Index (750;800;695;740) | Rirededge (750;800;695;740) | ((R750 - R800) / (R695 - R740)) - 1 | Gitelson et al., 2003 |
| 185 | Red Edge Triangular Vegetation Index (750;730;550;700;670) | RTVI (750;730;550;700;670) | (100 * ((R750 - R730) - 10 * (R750 - R550))) * (√(R700 / R670)) | Chen and Cihlar, 1996 |
| 186 | Red Edge Vegetation Stress Index (714;752;733) | RVSI (714;752;733) | (R714 + R752) / (2 - R733) | Merton, 1998 |
| 187 | Red Green Index (690;550) | RGI (690;550) | R690 / R550 | Zarco-Tejada et al., 2005 |
| 188 | Red Green Index (695;554) | RGI (695;554) | R695 / R554 | Gamon and Surfus, 1999 |
| 189 | Renormalized Difference Vegetation Index (800;670) | RDVI (800;670) | (R800 - R670) / (√(R800 + R670)) | Rougean and Breon, 1995 |
| 190 | Renormalized Difference Vegetation Index (880;673) | RDVI (880;673) | √(((R880 - R673) / (R880 + R673)) * (R880 - R673)) | Rougean and Breon, 1995 |
| 191 | Shortwave Infrared Normalized Difference Residue Index (2210;2260) | SINDRI (2210;2260) | (R2210 - R2260) / (R2210 + R2260) | Serbin et al., 2009 |
| 192 | Simple Ratio (1070;1340) | SR (1070;1340) | R1070 / R1340 | Rodríguez-Pérez et al., 2007 |
| 193 | Simple Ratio (415;685) | SR (415;685) | R415 / R685 | Read et al., 2002 |
| 194 | Simple Ratio (415;710) | SR (415;710) | R415 / R710 | Read et al., 2002 |
| 195 | Simple Ratio (440;685) | SR (440;685) | R440 / R685 | McMurtrey et al., 1994 |
| 196 | Simple Ratio (525;685) | SR (525;685) | R525 / R685 | McMurtrey et al., 1994 |
| 197 | Simple Ratio (533;537) | SR (533;537) | R553 / R537 | Tian et al., 2013 |
| 198 | Simple Ratio (545;538) | SR (545;538) | R545 / R538 | Tian et al., 2013 |
| 199 | Simple Ratio (550;670) | SR (550;670) | R550 / R670 | Carter, 1994 |
| 200 | Simple Ratio (678;1070) | SR (678;1070) | R678 / R1070 | Rodríguez-Pérez et al., 2007 |
| 201 | Simple Ratio (678;880) | SR (678;880) | R678 / R880 | Rodríguez-Pérez et al., 2007 |
| 202 | Simple Ratio (685;655) | SR (685;655) | R685 / R655 | Zarco-Tejada et al., 2003a |
| 203 | Simple Ratio (690;655) | SR (690;655) | R690 / R655 | Zarco-Tejada et al., 2003a |
| 204 | Simple Ratio (700;670) | SR (700;670) | R700 / R670 | McMurtrey et al., 1994 |
| 205 | Simple Ratio (700) | SR (700) | 1 / R700 | Gitelson et al., 1999 |
| 206 | Simple Ratio (750;705) | SR (750;705) | R750 / R705 | Sims and Gamon, 2002 |
| 207 | Simple Ratio (752;690) | SR (752;690) | R752 / R690 | Gitelson and Merzlyak, 1997 |
| 208 | Simple Ratio (780;740) | SR (780;740) | R780 / R740 | Mistele and Schmidhalter, 2010 |
| 209 | Simple Ratio (800;670) | SR (800;670) | R800 / R670 | Jordan, 1969 |
| 210 | Simple Ratio (801;550) | SR (801;550) | R801 / R550 | Daughtry et al., 2000 |
| 211 | Simple Ratio (801;670) | SR (801;670) | R801 / R670 | Daughtry et al., 2000 |
| 212 | Simple Ratio (830;660) | SR (830;660) | R830 / R660 | Jordan, 1969 |
| 213 | Simple Ratio (845;665) | SR (845;665) | R845 / R665 | Broge and Mortensen, 2002 |
| 214 | Simple Ratio (900;680) | SR (900;680) | R900 / R680 | Peñuelas and Filella, 1998 |
| 215 | Simple Ratio (927;687) | SR (927;687) | R927 / R687 | Thenkabail et al., 2000 |
| 216 | Simple Ratio (960;950) | SR (960;950) | R960 / R950 | Dzikiti et al., 2010 |
| 217 | Simple Ratio (990;720) | SR (R990;R720) | R990 / R720 | Yao et al., 2010 |
| 218 | Simple Ratio Pigment Index (430;680) | SRPI (430;680) | R430 / R680 | Peñuelas et al., 1993b |
| 219 | Simple Ratio Water Index (1350;870) | SRWI (1350;870) | R1350 / R870 | Rodríguez-Pérez et al., 2007 |
| 220 | Simple Ratio Water Index (858;1240) | SRWI (858;1240) | R858 / R1240 | Zarco-Tejada et al., 2003b |
| 221 | Simple Ratio Water Index (860;1240) | SRWI (860;1240) | R860 / R1240 | Zarco-Tejada et al., 2003b |
| 222 | Simple Ratio Water Index (880;1265) | SRWI (880;1265) | R880 / R1265 | Rodríguez-Pérez et al., 2007 |
| 223 | Single Band (460) | SB (460) | R460 | Curran, 1989 |
| 224 | Single Band (655) | SB (655) | R655 | Richter et al., 2009 |
| 225 | Soil Adjusted Vegetation Index 1 (800;670) | SAVI 1 (800;670) | (1 + 0.5) * (R800 - R670) / (R800 + R670 + 0.5) | Huete, 1988 |
| 226 | Soil Adjusted Vegetation Index 2 (800;670) | SAVI 2 (800;670) | R800 / (R670 + (0.0183 / 1.2344)) | Major et al., 1990 |
| 227 | Soil Adjusted Vegetation Index (807;736) | SAVI (807;736) | (1 + 0.5) * (R807 - R736) / (R807 + R736 + 0.5) | Huete, 1988 |
| 228 | Soil Adjusted Vegetation Index (830;660) | SAVI (830;660) | (1 + 0.5) * (R830 - R660) / (R830 + R660 + 0.5) | Huete, 1988 |
| 229 | Spectral Polygon Vegetation Index (800;670;530) | SPVI (800;670;530) | 0.4 * (3.7 * (R800 - R670)) - 1.2 * √((R530 - R670) * (R530 - R670)) | Vincini et al., 2006 |
| 230 | Spectral Polygon Vegetation Index (800;670;550) | SPVI (800;670;550) | 0.4 * (3.7 * (R800 - R670) - 1.2 * \|(R550 - R670)\|) | Vincini et al., 2006 |
| 231 | Structure Independent Pigment Index (800;440;680) | SIPI (800;440;680) | (R800 - R440) / (R800 - R680) | Peñuelas et al., 1995 |
| 232 | Structure Independent Pigment Index (800;445;680) | SIPI (800;445;680) | (R800 - R445) / (R800 + R680) | Peñuelas et al., 1995 |
| 233 | Structure Independent Pigment Index (800;450;650) | SIPI (800;450;650) | (R800 - R450) / (R800 - R650) | Peñuelas et al., 1995 |
| 234 | Structure Independent Pigment Index (800;450;680) | SIPI (800;450;680) | (R800 - R450) / (R800 - R680) | Peñuelas and Inoue, 1999 |
| 235 | TCARI / OSAVI (700;670;550;800;670) | TCARI / OSAVI (700;670;550;800;670) | 3 * ((R700 - R670) - ((0.2 * (R700 - R550)) * (R700 / R670))) / (1 + 0.16) * (R800 - R670) / (R800 + R670 + 0.16) | Haboudane et al., 2002 |
| 236 | Transformed Chlorophyll Absorbtion Ratio (700;600;550;850;670) | TCARI (700;600;550;850;670) | 3 * ((R700 - R600) - ((0.2 * (R700 - R550)) * (R700 / (R850 + R670)))) | Haboudane et al., 2002 |
| 237 | Transformed Chlorophyll Absorbtion Ratio (700;670;550) | TCARI (700;670;550) | 3 * ((R700 - R670) - ((0.2 * (R700 - R550)) * (R700 / R670))) | Haboudane et al., 2002 |
| 238 | Transformed Chlorophyll Absorbtion Ratio (750;705;550) | TCARI (750;705;550) | 3 * ((R750 - R705) - ((0.2 * (R750 - R550)) * (R750 / R705))) | Wu et al., 2008a |
| 239 | Transformed Soil Adjusted Vegetation Index (800; 670) | TSAVI (800; 670) | (1.2344 * (R800 - (1.2344 * R670) - 0.0183)) / ((1.2344 * (R800 + R670)) - (1.2344 * 0.0183)) | Baret et al., 1989 |
| 240 | Transformed Soil Adjusted Vegetation Index (875;680) | TSAVI (875;680) | (1.062 * (R875 - (1.062 * R680) - 0.022)) / (R680 + (1.062 * R875) - (1.062 * 0.022) + (0.08 * (1 + (1.062 * 1.062)))) | Rondeaux et al., 1996 |
| 241 | Triangular Vegetation Index (750;550;670) | TVI (750;550;670) | 0.5 * (120 * (R750 - R550) - 200 * (R670 - R550)) | Broge and Leblanc, 2001 |
| 242 | Triangular Vegetation Index (758;554;674) | TVI (758;554;674) | 0.5 * (120 * (R758 - R554) - 200 * (R674 - R554)) | Rodríguez-Pérez et al., 2007 |
| 243 | Vegetation Index (800;694) | VI (800;694) | (R800 / R694) - 1 | Viña, 2003 |
| 244 | Vegetation Stress Ratio (725;702) | VSR (725;702) | R725 / R702 | White et al., 2008 |
| 245 | Vogelmann (734;747;715;720) | Vogelmann (734;747;715;720) | (R734 - R747) / (R715 + R720) | Vogelmann, et al., 1993 |
| 246 | Vogelmann (734;747;715;726) | Vogelmann (734;747;715;726) | (R734 - R747) / (R715 + R726) | Vogelmann, et al., 1993 |
| 247 | Vogelmann (740;720) | Vogelmann (740;720) | R740 / R720 | Vogelmann, et al., 1993 |
| 248 | Water Index (900;970) | WI (900;970) | R900 / R970 | Peñuelas et al., 1993a |
| 249 | Water Index (950;900) | WI (950;900) | R950 / R900 | Peñuelas et al., 1993b |
| 250 | Water Index (970;900) | WI (970;900) | R970 / R900 | Peñuelas et al., 1993a |
| 251 | Weighted Difference Vegetation Index (800;670) | WDVI (800;670) | R800 - (1.2344 * R670) | Clevers, 1989 |
| 252 | Weighted Difference Vegetation Index (830;660) | WDVI (830;660) | R830 - (1.06 * R660) | Clevers, 1989 |
| 253 | WI / NDVI (970;900;800;680) | WI / NDVI (970;900;800;680) | (R970 / R900) / ((R800 - R680) / (R800 + R680)) | Peñuelas and Inoue, 1999 |
| 254 | Yellow Red Chlorophyll Absorption Ratio (600;680) | YCAR (600;680) | R600 / R680 | Schlemmer et al., 2005 |
| 255 | Zarco and Miller (750;710) | ZM (750;710) | R750 / R710 | Zarco-Tejada et al., 2001 |
